# Practical Applications of a Set-Based Camera Deployment Methodology

**DOI:** 10.3390/s24010111

**Published:** 2023-12-25

**Authors:** Edward Parrott, Joshua K. Pickard, Rickey Dubay

**Affiliations:** 1 Department of Mechanical Engineering, University of New Brunswick, Fredericton, NB E3B 5A3, Canada; dubayr@unb.ca; 2 Eigen Innovations Inc., Fredericton, NB E3B 1S1, Canada; josh.pickard@eigen.io

**Keywords:** quality inspection, interval analysis, camera models, intelligent inspection, constraint satisfaction, advanced manufacturing

## Abstract

This work establishes a complete methodology for solving continuous sets of camera deployment solutions for automated machine vision inspection systems in industrial manufacturing facilities. The methods presented herein generate constraints that realistically model cameras and their associated intrinsic parameters and use set-based solving methods to evaluate these constraints over a 3D mesh model of a real part. This results in a complete and certifiable set of all valid camera poses describing all possible inspection poses for a given camera/part pair, as well as how much of the part’s surface is inspectable from any pose in the set. These methods are tested and validated experimentally using real cameras and precise 3D tracking equipment and are shown to accurately align with real imaging results according to the hardware they are modelling for a given inspection deployment. In addition, their ability to generate full inspection solution sets is demonstrated on several realistic geometries using realistic factory settings, and they are shown to generate tangible, deployable inspection solutions, which can be readily integrated into real factory settings.

## 1. Introduction

This paper performs a set-based synthesis of complete inspection pose solutions for arbitrary 3D geometries that account for a wide range of non-linearities present in real cameras. This includes realistic constraint parameter generation methods, a modified constraint satisfaction algorithm based on interval branch-and-bound techniques, methods to test for visibility in the presence of lens distortion, and a multi-camera deployment recommendation algorithm. These combine to create a practically usable algorithm for the certifiable set-based synthesis of a complete set of inspection poses for arbitrary 3D meshes representing real manufactured parts based on specific stopping criteria. The results are validated experimentally to confirm that they do indeed generate reliable and accurate estimations of a given camera’s inspection capabilities from a given pose interval vector, and then the results are presented for simulations of realistic industrial inspection scenarios.

## 2. Preliminary Theory and Literature Review

### 2.1. Problem Terminology

In order to aid in the reader’s understanding of the existing literature and the problem definition, it is useful to first define some key terms, which will be used throughout.

First, the model must be defined as the 3D geometric representation of the part. It is standard practice to use triangular mesh models [1,2,3,4], which represent a part as a triangular mesh with edges, faces, and vertices defining the 3D geometric structure of the part and its surfaces [5]. Each vertex is a 3D point, and the complete set of them makes up each point on the part’s surface. Each facet is then represented as a triangular surface bounded by three vertices and the edges connecting them, along with an outward-facing unit normal vector [1,2,3,4]. The tessellation of all facets creates the 3D geometry of the entire part. We will also define the “facets of interest”, or “foi”, here, which are simply the subset of facets on the part’s surface that the given camera must inspect and for which the sets of inspection poses will be defined according to the sets of inspection constraints.

Next, the inspection task must be defined [6] as the set of measurements that must be conducted on a part with a known model. As such, this work will consider the inspection task as the surfaces of a part (i.e., the facets of interest) whose imaging poses need to satisfy inspection constraints defined by the camera used for imaging. This task will be planned according to a 3D model of the part and a corresponding set of inspection constraints. Inspection constraints are derived according to camera parameters and serve to constrain the region in front of the camera in which a captured image will meet the inspection requirements.

With the model and inspection task now defined, the inspection space can be discussed. Using certified set-based methods, the methods presented herein generate a complete set of valid inspection pose vectors for each facet of interest on the part. The complete set of these pose vectors defines a 6D region around the part wherein any included camera pose will satisfy the inspection task constraints for at least one facet of interest, i.e., the given facet will be properly inspected. This region will be called the inspection space.

With the model and inspection task now defined, the inspection space can be discussed. In the simplest terms, the inspection space is the set of valid solutions to the inspection task for the model. This is generated herein using certified set-based methods to determine the full set of 6D pose vectors for which at least one facet of interest can be imaged successfully according to the inspection constraints.

The final term to define is the sensor, which is the device used to carry out the inspection task. In this context, it is an optical digital camera. While other sensor applications could be explored in future work using analogous methods with slightly modified inspection constraints, this research focuses on visual inspection, and as such, only optical cameras will be considered. The terms “camera” and “sensor” may thus be considered synonymous in the context of this paper.

### 2.2. Summary of the Existing Literature

The problem of inspection camera pose generation and deployment has existed in its current state since the mid-1990s [6,7,8]. Early methods focused on feature detection and inspection, which spawned the modern sensor deployment problem. These methods established the two key pillars of the inspection problem: the accurate identification and representation of the relevant sensor and object models [7]. Of the two, the most important is establishing an adequate sensor model, and subsequently, properly deriving the constraints these sensor characteristics impose on the inspection task.

The most basic model of the sensor seen in the literature is the pinhole camera model [3,4,6,9,10]. While this model is often considered accurate enough in many situations [9], in the context of this research, it does not sufficiently describe sensor behaviour. Thus, this work will build upon the basis established in [11] and use the standard thin lens model, which assumes an infinitesimally thin lens with a finite aperture diameter [12]. It should be noted that while there are more complex models [1,7,12,13] in the literature, their additional complexity is not required in the scope of this research.

**Figure 1:**
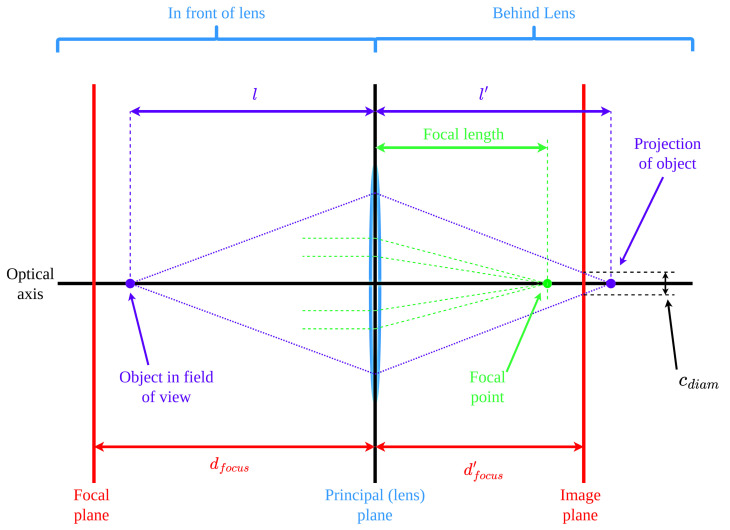
Thin lens model.

The principal diagram of the thin lens model is presented in Figure 1. The key parameters therein are the focal length *f*, the $f_{stop}$ number, the focal distance $d_{focus}$, and the blur circle diameter $c_{diam}$. These parameters are explained in detail in [11].

It was quickly established in the early literature that there are three main criteria required to adequately satisfy inspection task requirements [1,2,3,4,7]. They are that the inspection surfaces must be visible, the inspection surfaces must be imaged with suitably low blur, and the image resolution must be sufficient for the inspection task.

Methods to satisfy these constraints in camera deployments have continuously evolved. Initial methods placed the sensor on the surface of virtual hemispheres [6,7,14] surrounding the model to make sure the camera was always at a suitable distance and that the camera was oriented properly. Some other methods suggested ideas such as using sampled point clouds and advanced statistical filtering to place the camera and test for occluding geometry [15]. These early methods all typically used “generate and test” methodologies in which deployments are proposed according to informal heuristics and iteratively improved upon based on their performance [7,16]. These methods are no longer the standard as they are generally inefficient and unreliable.

With the advent of greater computing power, there was a notable shift away from qualitative “generate and test” methods toward more quantitative “synthesis” methods. Synthesis techniques [1,2,3,4,17,18,19,20,21,22] express inspection constraints as mathematical relationships between the sensor pose and inspection features so as to quantify their relationships within the context of the inspection task. Expressing the constraints in quantitative terms allows for them to be used in modern optimization routines, upon which most modern synthesize approaches are based. In the literature, we see recursive solvers [1], hierarchical genetic algorithms [2], artificial neural networks with fuzzy inference systems [17,18,19], Parisian algorithms [20], and advanced heuristic convex optimization methods, such as genetic algorithms or particle swarm optimization [3,4,23]. These approaches have all shown some degree of promise; however, they are all restricted by the fact that they generate unique discrete solutions, that they cannot guarantee repeatability, and that they are unable to account for real-world uncertainties in both modelling and deployment.

This gap in the literature is initially addressed in [11] by re-framing the context of the problem into a set-based framework and applying set- and interval analysis-based solving methods to rigorously and repeatably synthesize continuous solution sets that are robust to modelling and deployment uncertainties. However, that work is limited by only being able to synthesize solution sets for single facets of interest. This research uses a new solver method to allow for the easy intersection of multiple solution sets, which allows for the easy synthesis and optimization of solutions with multiple sensors with different intrinsic parameters for sets of multiple facets of interest.

### 2.3. Problem Definition

The problem that this research addresses is that of synthesizing a complete and certifiable set of possible inspection poses for the machine vision-based quality inspection of a manufactured part represented as a tessellated 3D mesh. By using set-based solvers and interval analysis mathematical frameworks, realistic camera models and their associated intrinsic parameters, and defining a set of multiple inspection facets, the method contained herein bridges the gap between existing theory [2,3] and practical industrial methods.

The fundamental process for solving camera deployment solutions involves the 6D search space about the part being iteratively subdivided according to interval analysis-based branch and bound algorithms [24,25,26], with each subdivision being tested to see if it satisfies the requisite constraints to be considered a valid pose solution for a given facet or set of facets. Constraints can be considered as either task constraints or sensor constraints. These are differentiated by whether the constraint parameters are informed by elements of the task itself or are dependent on sensor extrinsic and intrinsic parameters. The key task constraints considered are:

1. Is the set of facets of interest in front of the camera from a given set of poses?
2. Is the camera an appropriate distance from the set of facets of interest?
3. Are the facets of interest occluded by any other geometry?
4. Is the camera oriented such that all facets of interest are within its field of view (FOV)?

Subsequently, the key sensor constraints considered are:

1. Does the set of viewing angles allow for sufficient imaging resolution?
2. Are the facets of interest adequately in focus according to the blur constraints? Are the depth of field (DOF) limits appropriate based on camera parameters?
3. Do all facets of interest appear in the captured image, even in the presence of real lens distortions?

The algorithm established herein defines a novel modified constraint satisfaction algorithm to allow for the use of multiple facets of interest. It allows for the generation of constraint parameter-calibrated industrial cameras and accounts for camera extrinsic/intrinsic parameters, along with the inherent uncertainty in each. It also proposes a basic multi-camera deployment recommendation algorithm in order to use the generated pose solution sets to generate realizable camera deployment networks.

It also should be noted that the methods proposed herein shift the focus of the research from the problem of identifying the single optimal camera deployment solution from previous works to identifying the complete set of any and all valid solutions. This complete set of solutions can then be used as a tool to attempt to identify the optimal deployment, but identifying optimal solutions is not the primary goal of the constraint satisfaction solvers described in the following sections.

### 2.4. Interval Analysis Methods

This research solves a complete set of valid inspection poses by applying interval analysis methods. These methods create extensions to standard mathematical operations by using interval values instead of discrete numbers [25]. By treating numbers as intervals, one can compensate for measurement and rounding errors in calculations [25]. This allows interval-based constraint satisfaction algorithms to produce sets of continuous ranges of solutions that are guaranteed to contain valid solutions to given problems. For a complete summary of interval methods, as well as their application to constraint satisfaction problems, see [24,25,26,27]. For their application to this particular problem, see [11].

Intervals are defined in this context according to

(1) $$\left[x\right]=\left[\underline{x},\bar{x}\right]=\left\{x\in R|\underline{x}\leq x\leq \bar{x}\right\}$$

where $\underline{x}$ and $\bar{x}$ are the lower and upper bounds of the interval.

## 3. Constraint Satisfaction Algorithm

The case of a single-facet pose solver algorithm using standard branch-and-bound methods [24,26,28] presented in [11] solved the poses by iterating over position constraints then orientation constraints to refine a single facet’s inspection space up to a given resolution. However, this method is not suitable for the added complexity of generating pose solutions for a multi-facet mesh, and so a new constraint solver algorithm is proposed. Whereas a detailed breakdown of a single facet solver algorithm is presented in [11], the current work focuses on the more complex multi-facet solver.

The multi-facet formulation starts with a definition of a box. Each box is defined as an object whose attributes are a 6D interval vector describing its full set of valid poses, a classification as either valid for the facet of interest, partially valid for the facet of interest, or invalid for the facet of interest, and a unique ID number, along with the ID numbers of both its parent and child boxes (when a box is subdivided via bisection, the two resulting boxes are considered the children of the original parent box). The multi-facet case uses this as a starting point but adapts it and adds several new features that allow for a more efficient evaluation of the search space with multiple facets of interest. Boxes are organized in a hierarchical tree structure populated during bisection operations and are defined as objects with the following parameters:

1. A 6D pose interval vector;
2. A list of valid facets;
3. A list of boundary facets;
4. A unique ID key;
5. The ID key of the parent;
6. A Boolean value designating if the box is a leaf on the tree;
7. A Boolean value designating if the box is a boundary box;
8. The vector of interval vectors containing orientation angles for all valid facets;
9. The vector of interval vectors containing orientation angles for all boundary facets.

It should be noted that in this context, a valid facet is considered to be a facet of interest for which a given box satisfies all task and sensor constraints, and a boundary facet is a facet of interest for which a box contains a region that satisfies constraints but for which the entire box does not represent a completely valid solution. Additionally, a leaf on the tree is defined as a box which does not require full subdivision (this will be explored in greater detail in later sections), and a boundary box is one that will not be further subdivided and has no valid facets but some boundary facets.

**Figure 2:**
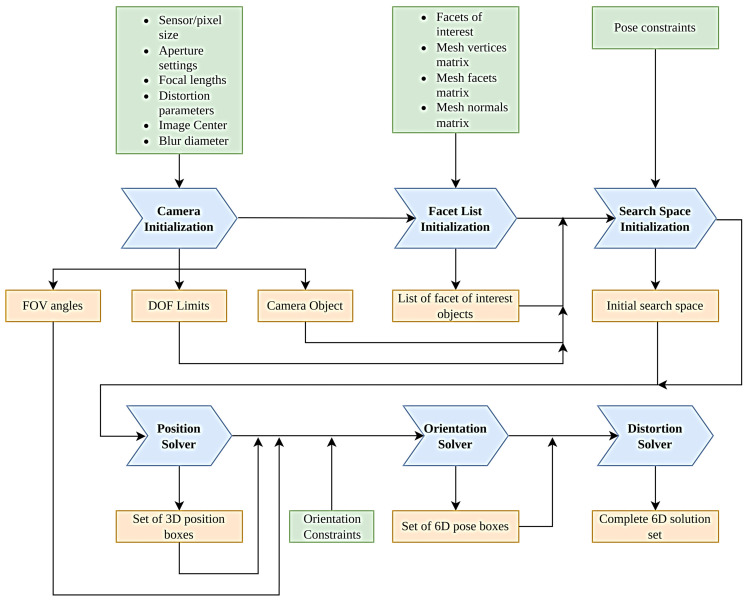
High-level structure of the solver algorithm.

The multi-facet algorithm retains the structure of first solving the x-y-z position intervals for the full set of boxes and then solving the valid orientation angles for each box established in [11], but it adds some additional complexities, including constraint generation from real camera intrinsics taking into account realistic camera models and an additional stage at the end testing for any facets that will be pushed out of the camera’s image by lens distortion effects. All of these will be explored in greater detail in later sections, but at a high level, the algorithm functions according to the flowchart presented in Figure 2.

### 3.1. Algorithm Initialization

The algorithm’s initialization involves pre-solving and pre-allocating as many solver parameters as possible before entering the main loop. Pre-allocated parameters include the camera’s intrinsic parameters, the camera matrix, the minimum and maximum depth of field values, the field of view angles, and the minimum required angles of incidence. This is carried out to reduce loop time by having as much prior information as possible instead of having to solve for important parameters inside the loop. The key parameters that are initialized at this stage are the camera object and constraints, the facet of interest objects, and the initial box.

#### 3.1.1. Camera Parameter Initialization

At the heart of the pose synthesis algorithm is the camera from which the poses will be derived. While detailed descriptions of camera models, parameters, and constraint derivations will be explored in greater detail in later sections, at this stage, it is important to know that the camera is defined as an object with the following parameters:

1. The field of view angles $\alpha_{h}$ and $\alpha_{v}$;
2. Available f-stop (aperture) settings;
3. The focal length components $f_{x}$ and $f_{y}$ and image center values $c_{x}$ and $c_{y}$ from the camera matrix;
4. Sensor size and pixel pitch;
5. Brown–Conrady lens distortion parameters;
6. Depth of field limits;
7. The maximum allowable image blur circle diameter, $c_{diam}$.

Most of these are specifications available directly from camera datasheets (field of view angles, f-stops, focal length, and sensor/pixel dimensions), but others must be solved or derived by the user. For instance, distortion parameters, image center values, and the *x* and *y* focal lengths must be derived by the user during camera calibration (they are usually used for image distortion correction algorithms, but they also inform the derivation of distortion constraints later on); the maximum blur circle diameter is specified by the user, and depth of field limits must be solved based on available f-stop values. For a detailed description of the camera matrix parameters and their derivations, see [29].

**Figure 3:**
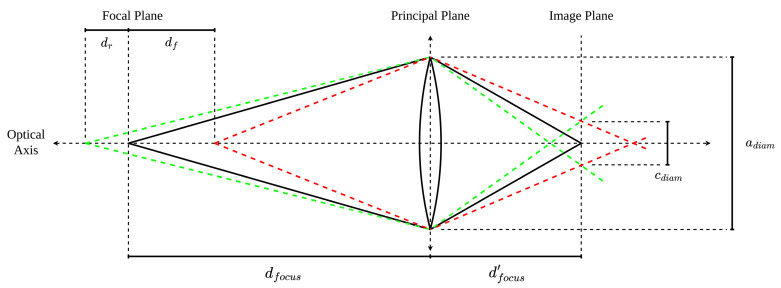
Thin lens model with DOF limits. The red and green lines denote the frant and rear limits of the depth of field.

In order to determine the acceptable distance range for an object to be imaged suitably sharply, the front and rear depth of field limits, $d_{f}$ and $d_{r}$, must be evaluated. Figure 3 expands on Figure 1 to show the DOF limits and their relationship to $c_{diam}$.

Calculating the DOF limits requires knowledge of the hyperfocal distance, $d_{HF}$, which represents the distance beyond which any objects will appear equally in focus on the image plane, regardless of their relative positions. It is defined as

(2) $$d_{HF}=\frac{f^{2}}{f_{stop}c_{diam}}+f$$

We must also determine our maximum allowable blur circle diameter, $c_{diam}$, which is defined as

(3) $$c_{diam}=a_{diam}\frac{l^{\prime }-d_{focus}^{\prime }}{l^{\prime }}$$

The DOF limits are then calculated [30] according to Equations (4) and (5),

(4) $$d_{f}=\frac{d_{HF}d_{focus}}{d_{HF}+\left(d_{focus}-f\right)}=\frac{d_{focus}c_{diam}f_{stop}\left(d_{focus}-f\right)}{f^{2}+c_{diam}f_{stop}\left(d_{focus}-f\right)}$$

(5) $$d_{r}=\frac{d_{HF}d_{focus}}{d_{HF}-\left(d_{focus}-f\right)}=\frac{d_{focus}c_{diam}f_{stop}\left(d_{focus}-f\right)}{f^{2}-c_{diam}f_{stop}\left(d_{focus}-f\right)}$$

The full acceptable distance interval for the image, $d_{img}$, is then defined in Equation (6) as

(6) $$\left[d_{img}\right]=\left[d_{focus}-d_{f},\;d_{focus}+d_{r}\right]$$

It should be noted that this approach assumes a fixed working distance for a lens, i.e., one that does not have variable focus settings. While this approach is fine in theory, in practice, most lenses have variable focus settings, and as such, it is overly simplistic and must be extended to model a variable-focus lens. Fortunately, the extension is not overly complex. The focus setting on a standard variable-focus lens is typically referred to as the working distance, or what has previously been named $d_{focus}$. To reiterate, this quantity is the distance at which a point will be perfectly projected onto the image plane with no blur. Most standard focus lenses have working distance settings from some minimum working distance (the smallest distance at which the lens can perfectly project a point onto the image plane), $d_{MW}$, up to infinity. In practice, though, the maximum setting is the hyperfocal distance. While $d_{HF}$ is a parameter that the user must derive, $d_{MW}$ is defined by the camera/lens manufacturer.

In order to then determine the appropriate $\left[d_{img}\right]$ interval for a real variable-zoom lens, $\left[d_{img}\right]$ is calculated for $d_{focus}=d_{MW}$ and $d_{focus}=d_{HF}$. The intersection of these two intervals is then taken, and the result is used as the final $\left[d_{img}\right]$ for the definition of minimum and maximum distance constraints in the solver algorithm. This process is used because it allows for certification of the fact that if all features fall within this $\left[d_{img}\right]$ interval, there is some zoom setting on the lens that will allow for all features to be imaged with acceptable focus.

**Figure 4:**
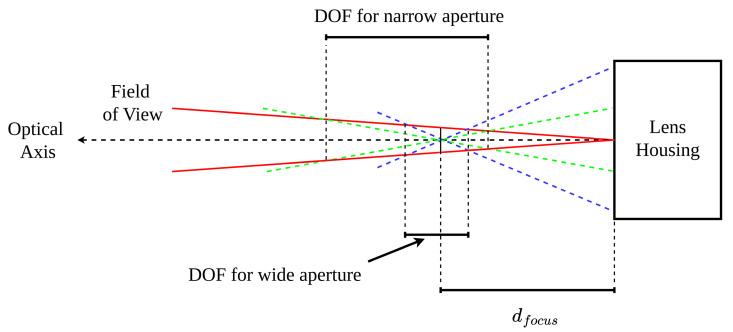
Effect of aperture diameter on DOF. Blue and green lines represent point projections for different aperture settings.

It is also important to briefly discuss the effect of $f_{stop}$ values on DOF intervals. As mentioned previously, the $f_{stop}$ value defines the relationship between focal length (a fixed quantity) and aperture diameter (a variable quantity). While on most practical lenses, the aperture diameter is technically continuously variable, the control for it is typically indexed to a standard set of values such that the amount of light entering through the lens increases/doubles by a factor of 2 at each setting. As such, it is often sufficient to calculate the discrete $\left[d_{img}\right]$ intervals at each standard setting in the lens range as opposed to calculating a continuous set of $\left[d_{img}\right]$ intervals. The aperture setting is typically selected based on the lighting conditions of the working environment but also by $\left[d_{img}\right]$ limits imposed by working environment geometry, as the $f_{stop}$ number also affects the available DOF. Figure 4 demonstrates how changing the aperture diameter changes the DOF for a fixed zoom setting.

By tracing a line from the aperture limits (in green for a narrow aperture and blue for a wide aperture) through the point on the optical axis at $d_{focus}$ and checking the points at which those lines intersect the nominal FOV frustum (in red), it is possible to see the $d_{img}$ interval for the given aperture diameter. Figure 4 thus demonstrates that a wider aperture setting results in a narrower DOF interval. This must then be taken into consideration along with environmental lighting conditions when selecting an aperture setting for a camera in a given deployment.

**Figure 5:**
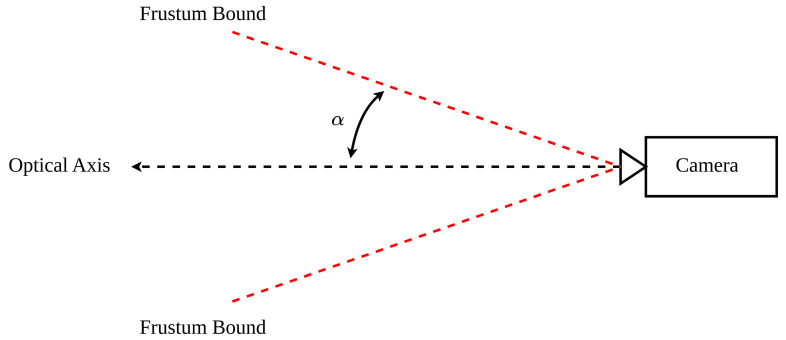
Field of view half-angle.

In the case where field of view angles are not explicitly included in camera documentation, it is possible to derive them as follows. They are commonly defined as half-angles, referred to as α, where the angle is defined as that between the optical axis and the plane defining the edge of the camera’s view frustum, as in Figure 5. The full angle would then be the angle between the two frustum bounding planes and is equal to 2α.

The horizontal ($\alpha_{h}$) and vertical ($\alpha_{v}$) FOV half-angles are calculated based on the camera’s focal length and corresponding sensor dimensions [31] according to

(7) $$\alpha_{h}=\arctan\left(\frac{w}{2f_{x}}\right)$$

(8) $$\alpha_{v}=\arctan\left(\frac{h}{2f_{y}}\right)$$

In these equations, *w* and *h* represent the sensor width and height in millimetres. Once the FOV angles have been solved, they are used to derive the allowable orientation intervals for box/facet pairs [11].

The authors would also like to acknowledge that for more complex sensors [32,33], more advanced parameter derivations would be required, but these are beyond the current scope of this research. In the case of the basic machine vision optical digital cameras discussed herein, these derivations and those in [11] are sufficient.

#### 3.1.2. Facet of Interest Initialization

The facet of interest initialization involves the specification of the facets of interest and then the initialization of several useful parameters. Facets of interest are initially specified by the user as those that require inspection for the given task, and the list of them is typically imported into the solver as a .csv file containing the IDs assigned to the facets of interest in the mesh by the mesh file. Once the list of facets of interest has been specified, each facet of interest is initialized as an object with the following parameters:

1. Facet ID;
2. Vertices in the mesh file;
3. Vertex positions;
4. Facet normal;
5. Facet geometric barycenter.

The facet ID, vertex indices and positions, and facet normals are all geometric quantities that can be found directly from the mesh file. It should be noted that all of these parameters are readily derived in most common geometry-processing libraries for 3D mesh models.

#### 3.1.3. Initial Camera Deployment Pose Interval

In order to begin the solver, an initial pose box must be declared that tightly bounds the region containing all possible solutions. This process defines a system of position constraints for each facet of interest, and that system is contracted using the HC4 and ACID contractors over these constraint systems (see [25,26] for details on interval contraction algorithms) over these constraints in order to find the region that most tightly bounds all valid solutions for that facet. This is carried out for each facet of interest, and then the union of all of those boxes is taken to find the region that is certified to contain all valid solutions for all facets of interest. Once the initial box has been solved, it has a unique ID assigned to it and has all facets of interest assigned to its list of boundary facets as potential candidates to be valid facets for future child boxes further along in the solver. It also has its parent ID listed as NULL as it is the first generation and has no parent.

### 3.2. Position Solver

Once the initial solver parameters have been established and the search space has been initialized, it is possible to begin evaluating the search space. At a high level, the position solver is given a list of boxes where each has a list of boundary facets of interest, and then for each boundary facet of interest, it evaluates its position constraints over the current test box (for a detailed breakdown of position constraints, see [11]). If the test box is found to fully satisfy all constraints for a given facet of interest, the occlusion condition is then tested according to the procedure derived in [11] for the test box/facet of interest pair. If no occlusion exists, the facet of interest is then considered a valid (observable) facet from the given position box, and it is removed from the boundary facet list and placed on the valid facet list. If at any point the test box is found to violate the constraints for any of its candidate boundary facets of interest, that facet of interest is removed from the boundary list and any future consideration for child boxes. Consequently, if a box is found to only partially satisfy a facet of interest’s constraints, that facet of interest is kept on the boundary facets list to be checked against future children of the current box. Once all position and occlusion constraints have been tested for all candidate facets of interest for the box, the algorithm then checks the box’s dimensions and boundary facets list. If the box’s largest dimension is above a given threshold and there are still boundary facets on its list, then the box is bisected into two children. The children are given unique identifiers, have the parent’s identifier attached to their parent parameter, inherit the parent’s valid and boundary facets list, and are pushed to the end of the list of boxes for the algorithm to test. However, if a box is found to have boundary facets remaining but is below the size threshold, it is classified as a boundary box and subjected to no further testing or bisection. If a box has no boundary facets remaining, it can be either classified as a leaf (if the number of valid facets is greater than zero) or an invalid box (which does not even partially satisfy the constraints for any facet of interest). In either scenario, the box will be removed from any further testing or bisection. This process repeats until all boxes have been eliminated or classified as boundary or leaf boxes.

#### 3.2.1. Position Constraints

The key position system constraints $C_{p}$ (see [11]) for a pose box [p] for a facet of interest are:

1. Does [p] intersect the facet of interest?
2. Is [p] an appropriate distance from the facet of interest?
3. Is [p] in front of the facet of interest?
4. Does [p] inspect the facet of interest from a suitable angle?

First, to test if [p] intersects the facet of interest, we consider the constraint

(9) $$\begin{array}{cc} \; & C_{f}\cap \left[p\right]\neq \emptyset ,\;\left[p\right]\;\mathrm{is}\;\mathrm{not}\;\mathrm{valid}\;\;\;\; \end{array}$$

(10) $$\begin{array}{cc} \; & C_{f}\cap \left[p\right]=\emptyset ,\;\left[p\right]\;\mathrm{satisfies}\;\mathrm{constraint}\;1,\;\mathrm{continue} \end{array}$$

To test that the camera is an appropriate distance from the box, the distance constraint

(11) $$C_{distance}={\left(\begin{array}{c} \left[x\right]\\ \left[y\right]\\ \left[z\right] \end{array}\right)}^{2}-{\left[c\right]}_{f}^{2}\subseteq \left(\begin{array}{c} \left[{\underline{d_{img}}}^{2},{\bar{d_{img}}}^{2}\right]\\ {}[{\underline{d_{img}}}^{2},{\bar{d_{img}}}^{2}]\\ {}[{\underline{d_{img}}}^{2},{\bar{d_{img}}}^{2}] \end{array}\right)$$

is evaluated.

Here, ${\left[c\right]}_{f}$ is the interval vector containing the valid solutions to $C_{f}$.

The constraint for testing whether a box [p] is in front of a facet is called the backface constraint and is represented by

(12) $$\left[p\right]=\left\{\begin{array}{cc} \mathrm{in}\;\mathrm{front}: & \mathrm{if}\;([p]-v)\cdot n>0\\ \mathrm{intersecting}: & \mathrm{if}\;0\in ([p]-v)\cdot n\\ \mathrm{behind}: & \mathrm{if}\;([p]-v)\cdot n<0 \end{array}\right.$$

Finally, to determine if the viewing angle is sufficiently large for the facet of interest to be inspectable, we use the following equation:

(13) $$\arccos\left({\left[p\right]}_{i}-f_{j_{c}}\right)\cdot n\leq \left(\frac{\pi }{2}-\theta_{v}\right)$$

where the constant $\theta_{v}$ is the minimum viewing angle and the facet’s geometric center is $f_{j_{c}}$.

If these constraints have been satisfied for a given box/facet pair, it is tested to determine the presence of any occluding geometry and the degree of any such occlusion. This is carried out by taking the convex hull of the box and facet and performing a mesh Boolean subtraction in which the part geometry (and any additional scene geometry) is subtracted from it. If the result of this operation is equivalent to the initial convex hull, there is no occlusion. If the hull has changed, the occlusion condition is tested by determining if there is a continuous path from any facet vertex to any box vertex along the surface of the resultant hull; if there is, the occlusion is only partial, and if not, the facet is fully occluded. See [11] for a complete derivation.

#### 3.2.2. Position Solver Algorithm

The position solver algorithm is presented in Algorithm 1.

Once all boxes have been evaluated and classified and their lists of valid facets have been established, the algorithm moves on to the orientation phase. It should be noted that the goal of this algorithm is not to solve for any optimal positions but rather to evaluate the search space and return the complete set of all regions therein that satisfy position inspection constraints for at least one facet of interest.
**Algorithm 1** Multi-facet position solver algorithm  1:Initialize empty list of unclassified position boxes $L_{u}$  2:Initialize empty list of classified position boxes $L_{c}$  3:Initialize search space by contracting position variables for each facet of interest to obtain the initial box [u]  4:Initialize boundary and valid facet lists $L_{b}$ and $L_{v}$ for [u]  5:Add [u] to the end of $L_{u}$  6:**while **$L_{u}\neq \emptyset$** do**  7:    Set ${\left[u\right]}_{i}$ at the end of $L_{u}$ as a test box  8:    **for** All facets $F_{b_{j}}$ in $L_{b_{i}}$ for ${\left[u\right]}_{i}$ **do**  9:        Evaluate position and occlusion constraints for $F_{b_{j}}$ over ${\left[u\right]}_{i}$10:        **if** Constraints are fully satisfied (facet $F_{b_{j}}$ if VALID for ${\left[u\right]}_{i}$) **then**11:           Pop $F_{b_{j}}$ from $L_{b_{i}}$ for ${\left[u\right]}_{i}$12:           Add $F_{b_{j}}$ to $L_{v_{i}}$ for ${\left[u\right]}_{i}$13:        **else if** Constraints are partially satisfied **then**14:           Facet $F_{b_{j}}$ remains in $L_{b_{i}}$ for ${\left[u\right]}_{i}$ for the next iteration15:        **else**16:           Pop $F_{b_{j}}$ from $L_{b_{i}}$ for ${\left[u\right]}_{i}$17:        **end if**18:    **end for**19:    **if** ($L_{b_{i}}$≠∅) ∧ ($L_{v_{i}}$≠∅) for ${\left[u\right]}_{i}$ **then**20:        **if** The widest interval in ${\left[u\right]}_{i}$ is greater than stopping criteria **then**21:           Bisect ${\left[u\right]}_{i}$ into ${\left[u\right]}_{i_{1}}$ and ${\left[u\right]}_{i_{2}}$22:           Assign $L_{b_{i}}$ and $L_{v_{i}}$ for ${\left[u\right]}_{i}$ to ${\left[u\right]}_{i_{1}}$ and ${\left[u\right]}_{i_{2}}$23:           Pop ${\left[u\right]}_{i}$ from $L_{u}$24:           Add ${\left[u\right]}_{i_{1}}$ and ${\left[u\right]}_{i_{2}}$ to the end of $L_{u}$25:        **else**26:           ${\left[u\right]}_{i}$ is LEAF27:           Pop ${\left[u\right]}_{i}$ from $L_{u}$ and add it to $L_{c}$28:        **end if**29:    **else if** $L_{b_{i}}$ = ∅ for ${\left[u\right]}_{i}$ **then**30:        **if** $L_{v_{i}}$ = ∅ for ${\left[u\right]}_{i}$ **then**31:           Pop ${\left[u\right]}_{i}$ from $L_{u}$32:        **else**33:           ${\left[u\right]}_{i}$ is LEAF34:           Pop ${\left[u\right]}_{i}$ from $L_{u}$ and add it to $L_{c}$35:        **end if**36:    **end if**37:**end while**38:Terminate the position solver and return $L_{c}$39:Pass $L_{c}$ to the orientation solver, and commence Algorithm 2

### 3.3. Orientation Solver

Once the full set of position boxes $L_{c}$ has been synthesized, along with their list of valid facets, their corresponding valid orientation intervals can be solved. It should be noted that each facet of interest has its orientation intervals solved for a given box according to the methods in [11], but there few additional steps in order to account for the box’s intervals having to represent a valid pose for multiple facets.

Initially, each facet on a given box’s valid and boundary lists has its valid orientation angles solved via the process outlined in [11], and the box’s orientation intervals are set as the union of all of those angle intervals. Next, the width of the box’s orientation intervals is checked, and if they are wider than the allowable maximum width determined by the camera’s FOV angles, the box is bisected along its widest orientation interval. Otherwise, the solver proceeds to check individual facets. It first checks the boundary facet intervals against the box interval, and if they intersect, the facet remains classified as a boundary facet, but if not, it is eliminated. For the valid facets, the solver checks if their intervals contain the midpoint of the box intervals. If they do, it remains a valid facet. If they do not but the intervals still both intersect, the facet is pushed to the boundary facet list, and if there is no intersection for one or both intervals it is eliminated altogether.

#### 3.3.1. Orientation Constraints

The orientation interval representation used herein is a ZXZ Euler rotation sequence [R([φ],[γ],[β])] as described in [11].

The first two components, [φ] and [γ], are defined as

(14) $$\left[\varphi \right]=\varphi_{nom}+{\left[\varphi \right]}_{offset}$$

(15) $$\left[\gamma \right]=\gamma_{nom}+{\left[\gamma \right]}_{offset}$$

where $\varphi_{nom}$ and $\gamma_{nom}$ are the angles corresponding to a rotation that orients the camera such that if it is located at the midpoint of the box, its axis will pass through the barycenter of the facet, as per [11]. Additionally, ${\left[\varphi \right]}_{offset}$ and ${\left[\gamma \right]}_{offset}$ are defined as

(16) $${\left[\varphi \right]}_{offset}=\left[-\alpha_{h}+\underline{\varphi_{left}},\alpha_{h}+\bar{\varphi_{right}}\right]$$

(17) $${\left[\gamma \right]}_{offset}=\left[-\alpha_{v}+\underline{\gamma_{down}},\alpha_{v}+\bar{\gamma_{up}}\right]$$

where $\varphi_{left}$, $\varphi_{right}$, $\gamma_{down}$, and $\gamma_{up}$ are how much the camera axis can rotate in any one direction while still keeping the facet entirely within its field of view for any position in ${\left(\left[x\right],\left[y\right],\left[z\right]\right)}^{T}$ [11].

The third component, [β], defines the allowable roll of the camera about its axis after rotations by [φ] and [γ]. To begin, we solve the interval projection of each facet vertex and [p] onto the camera’s image plane for any position in ${\left(\left[x\right],\left[y\right],\left[z\right]\right)}^{T}$ after rotations by [φ] and [γ]. We call the corresponding interval vectors representing these projections ${\left[d\right]}_{p_{i}}$, i=1,…,3 [11]. Then, we define the constraints

(18) $${\left[d\right]}_{p_{i}}>{\left(0\right)}_{3x1}$$

(19) $${\left[d\right]}_{{p_{i}}_{1:2}}<\left(\begin{array}{c} w\\ h \end{array}\right)$$

We can subsequently use the HC4 and ACID contractors [26] to contract the domain of [β] to a point where the constraints are satisfied [11]

#### 3.3.2. Orientation Solver Algorithm

The aforementioned process is summarized in Algorithm 2.

It should be noted that the goal of this algorithm is not to solve for any optimal positions but rather to evaluate the search space and return the complete set of all regions therein that satisfy the pose inspection constraints for at least one facet of interest.
**Algorithm 2** Multi-facet orientation solver algorithm  1:Import the position box list $L_{c}$, and rename it $L_{u}$  2:Initialize the boundary and valid facet lists $L_{b_{i}}$ and $L_{v_{i}}$ for $\left[u_{i}\right]$ in $L_{u}$  3:**for** All ${\left[u\right]}_{i}$ in $L_{u}$ **do**  4:    Set ${\left[u\right]}_{i}$ orientation intervals as empty  5:    **for** All facets $F_{v_{j}}$ in $L_{v_{i}}$ for ${\left[u\right]}_{i}$ **do**  6:        Solve the orientation intervals for $F_{v_{j}}$  7:        Store facet orientation intervals as 3-vector ${\left[a\right]}_{i}$ in list $L_{FacetAngles}$ for ${\left[u\right]}_{i}$  8:        Set ${\left[u\right]}_{i_{3:5}}$ = ${\left[u\right]}_{i_{3:5}}$∪${\left[a\right]}_{i}$  9:    **end for**10:**end for**11:Begin orientation refinement12:**for** All ${\left[u\right]}_{i}$ in $L_{u}$ **do**13:    Pop ${\left[u\right]}_{i}$ from $L_{u}$14:    **if** Widest interval in ${\left[u\right]}_{i_{3:5\;}}$ is wider than maximum allowable width **then**15:        Bisect ${\left[u\right]}_{i}$ into ${\left[u\right]}_{i_{1}}$ and ${\left[u\right]}_{i_{2}}$16:        Assign $L_{{FacetAngles}_{i}}$ from ${\left[u\right]}_{i}$ to ${\left[u\right]}_{i_{1}}$ and ${\left[u\right]}_{i_{2}}$17:        Add ${\left[u\right]}_{i_{1}}$ and ${\left[u\right]}_{i_{2}}$ to the end of $L_{u}$18:    **else**19:        **for** All $F_{v_{i}}$ in $L_{v}$ for ${\left[u\right]}_{i}$ **do**20:           **if ** ∅∉ ($L_{{FacetAngles}_{i}}$ ∩ ${\left[u\right]}_{i_{3:5}}$) **then**21:               **if** $L_{{FacetAngles}_{i}}$ ∋ midpoint(${\left[u\right]}_{i_{3:5}}$) **then**22:                   $F_{v_{i}}$ is VALID for ${\left[u\right]}_{i}$23:               **else**24:                   Pop $F_{v_{i}}$ from $L_{v}$ for ${\left[u\right]}_{i}$25:                   Add $F_{v_{i}}$ to the back of $L_{b}$ for ${\left[u\right]}_{i}$26:               **end if**27:           **else**28:               **if** Any component of ($L_{{FacetAngles}_{i}}$ ∩ ${\left[u\right]}_{i_{3:5}}$) ≠∅ **then**29:                   Pop $F_{v_{i}}$ from $L_{v}$ for ${\left[u\right]}_{i}$30:                   Add $F_{v_{i}}$ to the back of $L_{b}$ for ${\left[u\right]}_{i}$31:               **else**32:                   Pop $F_{v_{i}}$ from $L_{v}$ for ${\left[u\right]}_{i}$33:               **end if**34:           **end if**35:        **end for**36:        Add ${\left[u\right]}_{i}$ to $L_{k}$37:    **end if**38:**end for**39:Terminate the algorithm and return the solution list $L_{k}$

### 3.4. Lens Distortion Solver

**Figure 6:**
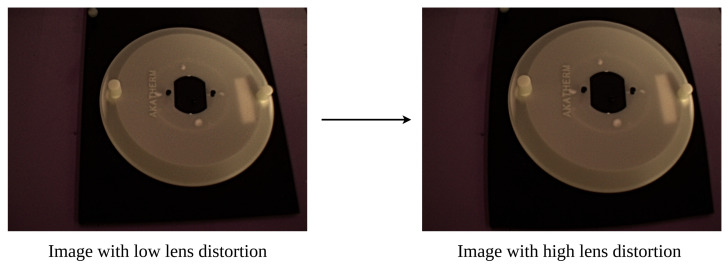
Example of how lens distortion could affect feature visibility.

Once the full set of 6D boxes has been synthesized, a final check must be performed before they can be considered the full solution set bound by real camera constraints. This final step is checking that all facets that are considered valid for any given box will still be present in a captured image from any pose within the set of valid poses even if the captured image is subject to lens distortion. Lens distortion is the warping of images due to the elliptical geometry of lenses and results in image points being shifted on the image plane away from the points at which they would be expected to project in an ideal pinhole projection. Figure 6 shows an exaggerated example of how lens distortion effects could push normally visible part features, such as the top edge of the sample part, out of frame.

In [29], the basic principles underlying how world coordinates are transformed into the camera’s coordinate system and subsequently projected into an image using a pinhole camera model are laid out. To summarize, an interval position in the world coordinate system, $\left[o_{W}\right]={\left(\left[x_{W}\right],\;\left[y_{W}\right],\;\left[z_{W}\right]\right)}^{T}$, is transformed into an interval projection in the camera’s relative coordinate system, $\left[o_{C}\right]={\left(\left[x_{C}\right],\;\left[y_{C}\right],\;\left[z_{C}\right]\right)}^{T}$ according to

(20) $$\left(\begin{array}{c} \left[x_{C}\right]\\ {}[{}_{C}]\\ {}[{}_{C}] \end{array}\right)=\left(\left[R\right]|\left[T\right]\right)\left(\begin{array}{c} \left[x_{W}\right]\\ {}[{}_{W}]\\ {}[{}_{W}] \end{array}\right)$$

Here, ([R]|[T]) is the camera’s homogeneous transformation matrix (see [29] for an in-depth discussion of the derivations of these quantities). However, at this point, the realistic projection model diverges from the previously established one. First, the *x* and *y* components of the image plane projected points are transformed according to

(21) $$\left[x_{img}^{\prime }\right]=\frac{\left[x_{C}\right]}{\left[z_{C}\right]}$$

(22) $$\left[y_{img}^{\prime }\right]=\frac{\left[y_{C}\right]}{\left[z_{C}\right]}$$

The quantities ${\left[r_{img}\right]}^{2}$ and [γ] must then be derived according to

(23) $${\left[r_{img}\right]}^{2}={\left[x_{img}^{\prime }\right]}^{2}+{\left[y_{img}^{\prime }\right]}^{2}$$

(24) $$\left[\gamma \right]=\frac{1+K_{1}{\left[r_{img}\right]}^{2}+K_{2}{\left[r_{img}\right]}^{4}+K_{3}r{{[}_{img}]}^{6}}{1+K_{4}{\left[r_{img}\right]}^{2}+K_{5}{\left[r_{img}\right]}^{4}+K_{6}{\left[r_{img}\right]}^{6}}$$

In Equation (24), the constants $K_{i}$ are the lens radial distortion constants from the standard Brown–Conrady distortion model. The projected $\left[x_{img}^{\prime }\right]$ and $\left[y_{img}^{\prime }\right]$ components are then further transformed according to

(25) $$\left[x_{img}^{\prime \prime }\right]=\left[\gamma \right]\left[x_{img}^{\prime }\right]+2P_{1}\left[x_{img}^{\prime }\right]\left[y_{img}^{\prime }\right]+P_{2}\left({\left[r_{img}\right]}^{2}+2{\left[x_{img}^{\prime }\right]}^{2}\right)$$

(26) $$\left[y_{img}^{\prime \prime }\right]=\left[\gamma \right]\left[y_{img}^{\prime }\right]+P_{1}\left({\left[r_{img}\right]}^{2}+2{\left[y_{img}^{\prime }\right]}^{2}\right)+2P_{2}\left[x_{img}^{\prime }\right]\left[y_{img}^{\prime }\right]$$

in which the constant $P_{i}$ is the Brown–Conrady tangential distortion coefficient. Both $P_{i}$ and $K_{i}$ are derived according to standard and well-defined camera calibration algorithms for machine vision. The projection of the point $\left[o_{C}\right]$ onto the image plane is then defined as    

(27) $$\left[\begin{array}{c} \left[x_{img}\right]\\ {}[y_{img}]\\ 1 \end{array}\right]=\left[\begin{array}{ccc} {}^{-1}{/}_{m_{x}} & 0 & c_{x}\\ 0 & {}^{-1}{/}_{m_{y}} & c_{y}\\ 0 & 0 & 1 \end{array}\right]\left[\begin{array}{ccc} f_{x} & 0 & 0\\ 0 & f_{y} & 0\\ 0 & 0 & 1 \end{array}\right]\left[\begin{array}{c} \left[x_{img}^{\prime \prime }\right]\\ {}[y_{img}^{\prime \prime }]\\ 1 \end{array}\right]$$

where $c_{x}$ and $c_{y}$ are the camera image centre coordinates, $m_{x}$ and $m_{y}$ are the pixel dimensions of the sensor, and $f_{x}$ and $f_{y}$ are the camera *x*- and *y*-axis focal lengths. While $m_{x}$ and $m_{y}$ are manufacturer-defined parameters, $c_{x}$, $c_{y}$, $f_{x}$, and $c_{y}$ must be derived according to standard camera calibration algorithms.

Any distortion calibration in this research is performed via Calib.io calibration routines using their proprietary camera calibration targets. The distortion calibration results were accurate to 5 decimal places.

#### Lens Distortion Constraints

In order to test that a given valid facet for a given box is fully contained in a captured image from any pose within the set, the bounding boxes containing the full set of possible projected positions of the facet’s vertices are derived using Equations (20)–(22). Their projections onto the image plane accounting for distortion are then solved by contracting over $\left[x_{img}\right]$ and $\left[y_{img}\right]$ according to equality constraints defined by Equation (27). This defines the box that is certified to most tightly bound the box containing the projection of the facet onto the image plane in the presence of lens distortions. Then, the image plane projection of this bounding box is compared to the area covered by the sensor on the image plane according to *w* and *h*, and if it falls entirely within the area covered by the sensor, it is kept as a valid facet. If it only partially intersects the sensor, it is pushed to the boundary facet list, and if the intersection is empty, then the facet is eliminated altogether from the box’s valid/boundary facet lists.

## 4. Deployment Recommendation Algorithm

Once the full set of valid poses has been synthesized via the solver algorithm (consisting of the position, orientation, and distortion solvers), a separate component is required to suggest the best possible deployments for a given camera. This is primarily due to the size of the final pose trees, as they often contain thousands of boxes, which in turn renders the human selection of pose boxes impractical. There are two primary algorithms developed for deployment selection: the single-camera deployment case and the multi-camera deployment case. Each works to maximize the number of facets covered by the recommended deployment. As such, the cost function can be defined as

(28) $$\mathrm{Maximize}\;n_{facets}$$

where $n_{facets}$ is the number of inspectable facets. It should be noted that the algorithms presented here are simply one possibility for solving the problem of generating individual deployments from the solution set; the deployment generation problem is, at this point, a maximum coverage problem for which graph theory researchers have generated a wide array of potential solutions that could also be deployed.

### 4.1. Single-Camera Deployment Recommendation

The single-camera deployment recommendation algorithm is an adapted greedy optimization algorithm in which the objective is simply the selection of boxes with the largest number of valid facets. To initialize the algorithm, the user first loads in the full set of pose intervals along with the facets of interest and then specifies how many recommendations are required.

Once those parameters are initialized, the algorithm scans the set of poses and selects the one with the largest number of associated valid facets as the first recommended deployment set. This pose interval is then eliminated from consideration for future recommendations. It then finds all other pose intervals that share five of six components as the currently recommended interval and eliminates them from consideration as well. This step is required because during orientation bisection, due to the orientation interval width requirements, orientation intervals are often bisected in a way that results in there being multiple boxes with identical [x]-[y]-[z] position vectors and two identical orientation intervals. This often presents functionally redundant solutions as these intervals often share identical valid facet lists. As such, they are removed to avoid redundancies in recommendations. This process then repeats with the remaining available poses until the required number of recommended deployments has been identified.

The algorithm is summarized in Algorithm 3.
**Algorithm 3** Single-camera deployment recommendation algorithm  1:Import full solution list $L_{s}$  2:Initialize recommended deployment list $L_{d}$ for the number of required deployments *n*  3:**for ***j* = 0 … (n−1) **do**  4:    Rank the solutions ${\left[s\right]}_{i}$ in $L_{s}$ by the number of valid facets  5:    Call the highest-ranked solution ${\left[s\right]}_{*}$  6:    Let deployment ${\left[d\right]}_{j}$ equal ${\left[s\right]}_{*}$  7:    Add ${\left[d\right]}_{j}$ to $L_{d}$  8:    Pop ${\left[s\right]}_{*}$ from $L_{s}$  9:    **for** All ${\left[s\right]}_{i}$ in $L_{s}$ **do**10:        **if** ${\left[s\right]}_{i_{0:2}}$ = ${\left[s\right]}_{{*}_{0:2\;}}$ **then**11:           **if** ${\left[s\right]}_{i_{3:5\;}}$ and ${\left[s\right]}_{{*}_{3:5\;}}$ have two identical components **then**12:               Pop ${\left[s\right]}_{i}$ from $L_{s}$13:           **end if**14:        **end if**15:    **end for**16:**end for**17:Let $L_{d}$ be the complete list of recommended solutions

### 4.2. Multiple-Camera Deployment Recommendation

The case of the multiple-camera recommendation algorithm is slightly more complex than the single-camera recommendation algorithm, but it is still essentially a modified greedy optimization at its core. The algorithm will be explained for a two-camera deployment case; however, the concepts are the same for any number of required cameras.

First, the algorithm scans the complete set of solutions $L_{s}$ and finds the box ${\left[s\right]}_{i}$ therein with the highest number of valid facets. Next, it scans the solution set for a second box, which contains the most valid facets not covered by the first choice. Since this example case only covers a two-camera deployment, this particular recommendation would then be considered complete. However, if more cameras were required, the process would continue finding subsequent maximum-remaining-coverage boxes until the required number had been selected.

When the algorithm then proceeds to subsequent deployment selections, it functions practically identically, except for a small extra first step in which the boxes already selected in previous recommendations are removed from consideration for future ones (along with any redundant boxes, as in the single-camera case). This is performed to encourage diversity in recommended deployment solutions.

The algorithm is summarized in Algorithm 4.
**Algorithm 4** Multi-camera deployment recommendation algorithm  1:Import the full solution list $L_{s}$  2:Initialize the recommended deployment list $L_{d}$ for the number of required deployments *n*  3:**for ***j* = 0 … (n−1) **do**  4:    Rank solutions ${\left[s\right]}_{i}$ in $L_{s}$ by the number of valid facets  5:    Call the highest-ranked solution ${\left[s\right]}_{*1}$  6:    Pop ${\left[s\right]}_{*1}$ from $L_{s}$  7:    **for** All ${\left[s\right]}_{i}$ in $L_{s}$ **do**  8:        **if** ${\left[s\right]}_{i_{0:2}}$ = ${\left[s\right]}_{{*1}_{0:2\;}}$ **then**  9:           **if** ${\left[s\right]}_{i_{3:5\;}}$ and ${\left[s\right]}_{{*1}_{3:5\;}}$ have two identical components **then**10:               Pop ${\left[s\right]}_{i}$ from $L_{s}$11:           **end if**12:        **end if**13:    **end for**14:    Rank the remaining solutions ${\left[s\right]}_{i}$ in $L_{s}$ by the number of valid facets not covered by ${\left[s\right]}_{*1}$15:    Call the highest-ranked solution ${\left[s\right]}_{*2}$16:    **for** All ${\left[s\right]}_{i}$ in $L_{s}$ **do**17:        **if** ${\left[s\right]}_{i_{0:2}}$ = ${\left[s\right]}_{{*2}_{0:2\;}}$ **then**18:           **if** ${\left[s\right]}_{i_{3:5\;}}$ and ${\left[s\right]}_{{*2}_{3:5\;}}$ have two identical components **then**19:               Pop ${\left[s\right]}_{i}$ from $L_{s}$20:           **end if**21:        **end if**22:    **end for**23:    Let the deployment pair (${\left[d\right]}_{j_{1}}$, ${\left[d\right]}_{j_{2}}$) = (${\left[s\right]}_{*1}$, ${\left[s\right]}_{*2}$)24:    Add (${\left[d\right]}_{j_{1}}$, ${\left[d\right]}_{j_{2}}$) to $L_{d}$25:**end for**26:Let $L_{d}$ be the complete list of recommended solutions

## 5. Case Study

### 5.1. Experimental Validation Study

In order to validate the practical applicability of the methods proposed herein, a series of practical experiments were conducted in order to assess the accuracy of the algorithm’s predicted results. These involved capturing images of a part from an industrial camera at various points from within a given interval box to compare with the algorithm’s predicted visibility results, both from the individual box vertices and for the box as a whole. Images were taken from individual box vertices because of the fact that is physically impossible to capture a continuous set of images from an interval vectors as the pose boxes are described. However, a unique property of interval analysis methods is that by taking the set intersection of a variety of solutions, one can create a composite solution showing the elements that are valid from all of the individual contributors. As such, by evaluating the results at the vertices and intersecting them, we can derive the set of valid solutions for the whole continuous pose box and compare this against the results simulated directly for the continuous box. Additionally, by taking images at discrete points, we can confirm the accuracy of the solutions by comparing our results against results computed for the same pose using tradiitonal rendering-based visibility methods. The combination of these factors along with the real images allows us to validate our results.

#### 5.1.1. Experimental Setup

The practical validation experimental setup involved using an injection-molded part and taking pictures of it with a camera whose extrinsic and intrinsic characteristics had been well-modelled and whose pose relative to the part could be tracked with a very high degree of accuracy.

The camera used was an Allied Vision Mako G-319C with an Edmunds Optics 8 mm megapixel fixed focal length lens. Using a 300 mm × 200 mm checkerboard calibration target with a 10 mm checker width and accompanying camera calibration software from Calib.io, along with the manufacturer’s specifications, the camera system’s parameters were derived and modelled. These parameters are summarized in Table 1.

**Figure 7:**
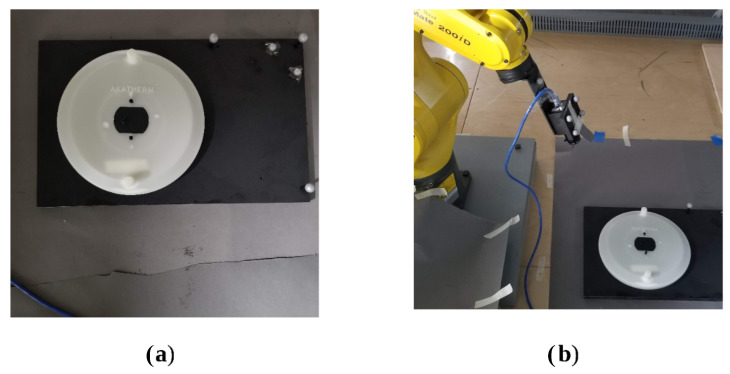
(**a**) Ground datum for validation testing with the part. (**b**) Full experimental setup.

In order to accurately track and adjust the camera pose relative to the part, the experiment made use of four OptiTrack Flex 13 motion capture cameras with the accompanying OptiTrack Motive (v. 2.3.2, Corvallis, OR, USA) motion capture and tracking software and a FANUC LR Mate 200iD industrial manipulator with a System R-30iB Mate Controller. After system calibration, we were able to achieve 3D tracking of all components with an accuracy of +/−1.4 mm. By making use of a specially constructed ground datum plate and camera mount for the robotic arm, which both incorporated infrared reflective motion capture markers along with OptiTrack’s proprietary Motive software, it was possible to track the camera and part’s 6D poses with sub-millimetre accuracy. Tracking their relative poses was then simply a matter of applying basic homogeneous transformations to the raw data from the OptiTrack software. The experimental setup is shown in Figure 7a,b.

#### 5.1.2. Experimental Results

The experiments to confirm the validity of the calculated validity results consisted of three stages: capturing images from a variety of poses, performing virtual renderings of these image/pose pairs (accounting for all real camera intrinsic/extrinsic properties) in order to confirm the validity of derived camera models, and the calculation of visibility results for the given part/pose and comparison of these results with the real image. Various sets of images and their associated calculated visibility results are presented in the following figures. All poses are defined as 6D vectors where the components are [*x*, *y*, *z*, φ, γ, and β], with the last three components being ZXZ Euler angles defining the camera’s orientation. The position values are measured in meters, and the orientation values are described in radians. The list of poses of the camera relative to the part is listed in Table 2.

The key constraint parameters derived from the camera parameters are listed in Table 3.

**Figure 8:**
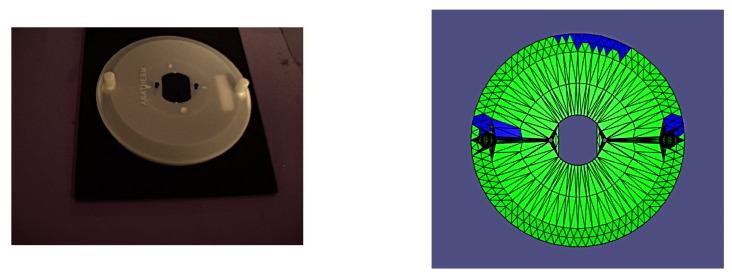
Real image of test pose 1 with evaluated inspection results.

**Figure 9:**
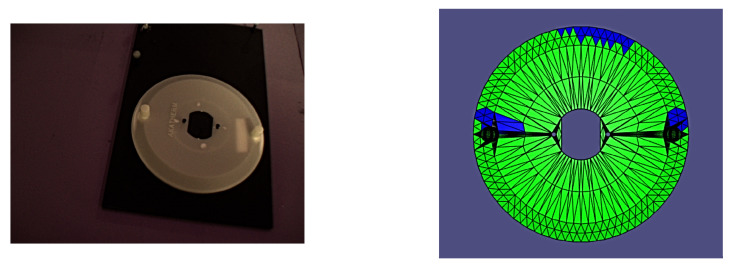
Real image of test pose 2 with evaluated inspection results.

**Figure 10:**
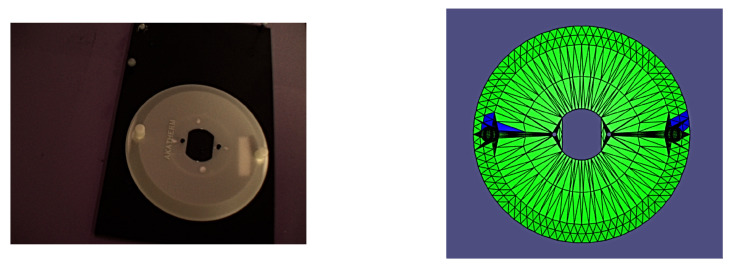
Real image of test pose 3 with evaluated inspection results.

**Figure 11:**
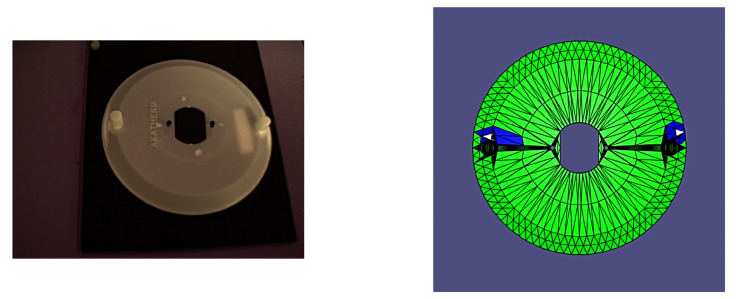
Real image of test pose 4 with evaluated inspection results.

**Figure 12:**
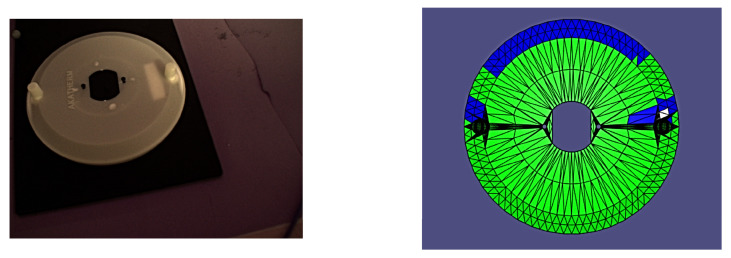
Real image of test pose 5 with evaluated inspection results.

**Figure 13:**
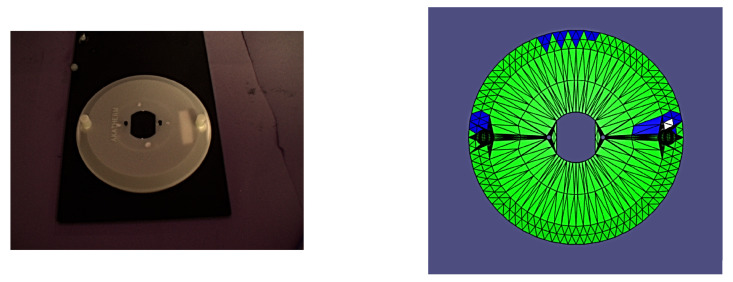
Real image of test pose 6 with evaluated inspection results.

**Figure 14:**
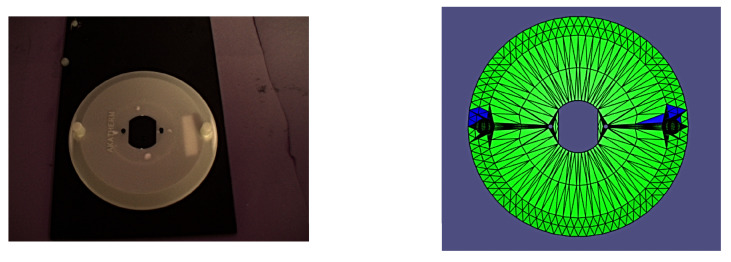
Real image of test pose 7 with evaluated inspection results.

**Figure 15:**
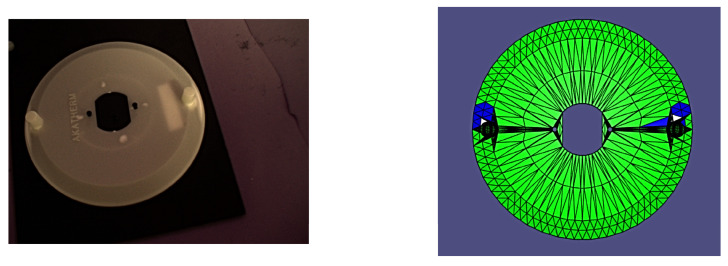
Real image of test pose 8 with evaluated inspection results.

It is worth noting that all of the poses presented herein are point poses, but this is due to it being impossible to visualize a continuous set of images from a continuous set of poses. In the following figures of results (Figure 8, Figure 9, Figure 10, Figure 11, Figure 12, Figure 13, Figure 14 and Figure 15), valid facets are denoted in green, partially valid facts are denoted in blue, and invalid facets are denoted in white.

Each simulation took approximately 26 min to process 9192 facets of interest on the mesh.

Each of the results from the discrete poses herein was found to agree with the expected visibility results using OpenGL rendering methods, and the intersection of all discrete solution sets agreed with the simulated results for the overall box. As such, we can consider the method to be valid based on the experimental study.

### 5.2. Full Camera Deployment Solutions

**Figure 16:**
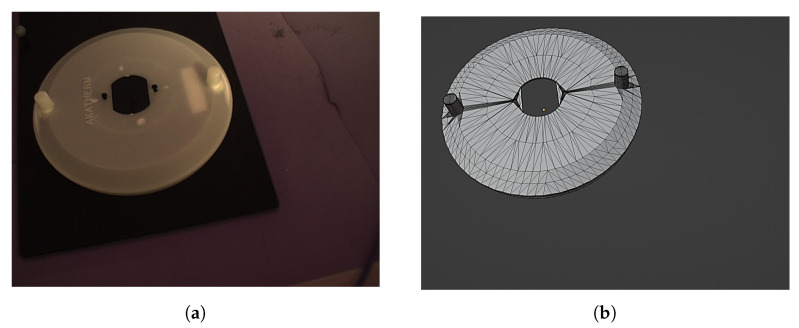
Sample part (**a**) and mesh representation (**b**).

A study was conducted in order to validate the complete algorithm and its components. The study was conducted using the sample part and its associated mesh representation presented in Figure 16.

The facets of interest were set to 100 facets on the top surface of the part. The camera parameters used were the same as those summarized in Table 1.

**Figure 17:**
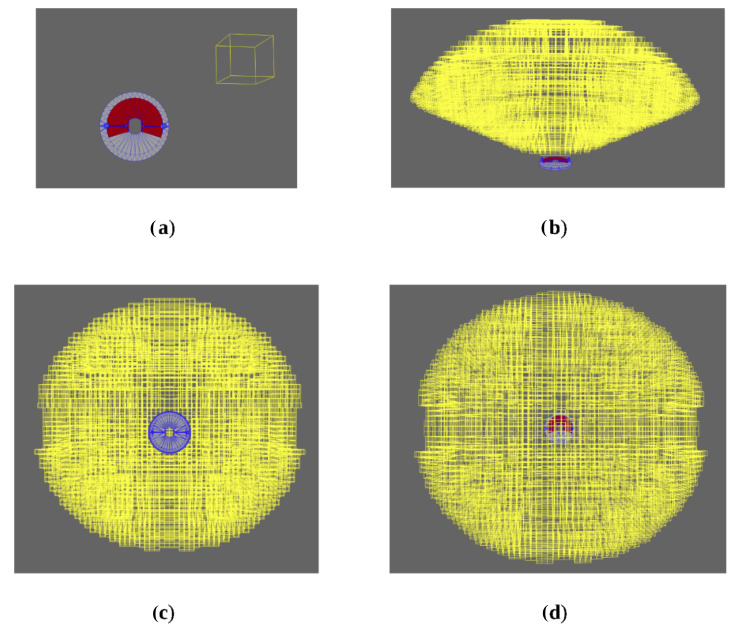
(**a**) Sample deployment box and associated visible facets from the set of facets of interest. (**b**) Full solution set, side view. (**c**) Full solution set, bottom view. (**d**) Full solution set, top view.

Figure 17a shows a sample single camera deployment, with all visible facets highlighted in red. The test generated 16,450 leaves, which are visualized in Figure 17b–d in space about the test part as yellow boxes.

In this figure, each yellow box represents one 6D interval vector belonging to the set of interval vectors that make up the complete solution. The 3 dimensions represent the *x*, *y*, and *z* components for the interval vectors, but each also contains the 3 orientation components as well. They are not visualized for the sake of not creating an overly messy visualization of the results, but the reader can refer to [11] for a further explanation of the orientation quantities and how they are calculated and presented.

**Figure 18:**
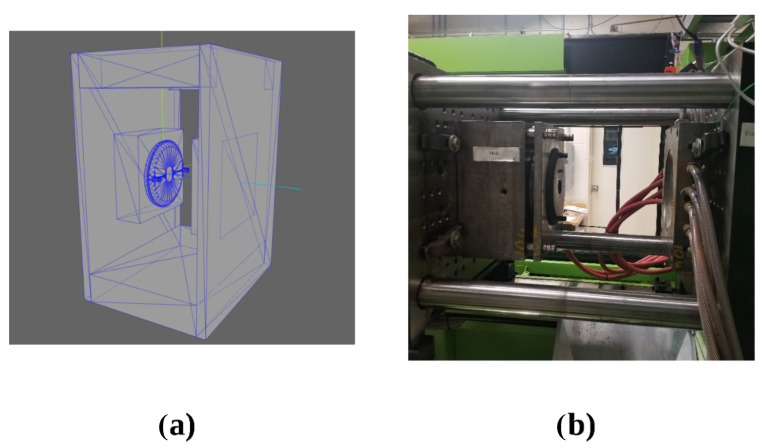
(**a**) Simulation of test part in injection moulding machine. (**b**) Real injection moulding machine with test part.

In order to further reinforce the real-world usability of the results, a test case was also performed to simulate the scenario in which an inspection deployment was desired for the test part used in previous experiments while it was being produced in an industrial injection moulding machine. Figure 18 shows the simulated geometry as well as the test part in a real injection machine.

The facets of interest are a selection of facets along the top surface of the part. The pose box width stopping criteria for this test was 0.1 m for position and 0.38 rads for orientation, with a 15-degree minimum viewing angle. The camera model was the Mako G-319C with an Edmunds Optics 8mm megapixel fixed focal length lens described in Section 5.1.1.

**Figure 19:**
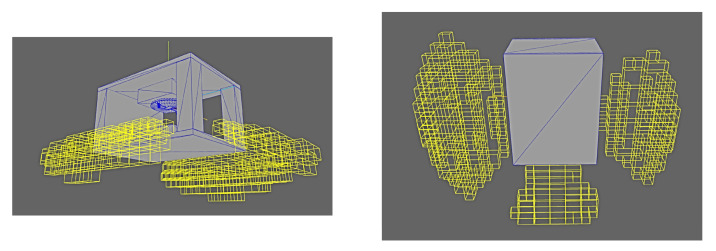
Results of deployment solver with test part in realistic factory setting.

**Figure 20:**
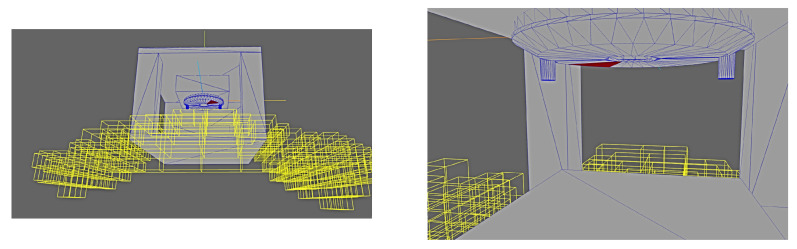
Results of deployment solver with test part in realistic factory setting.

The deployment results are shown from a variety of perspectives in Figure 19 and Figure 20. It can clearly be seen that the deployment solver avoids any poses which would be occluded either by the external geometry or any other features of the part itself (i.e., the protrusions on the top surface).

**Figure 21:**
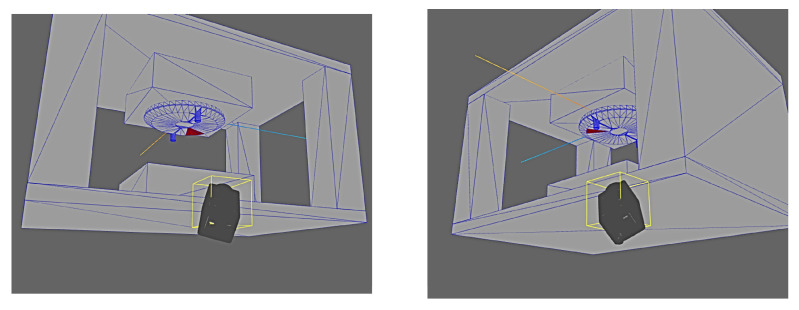
Results of deployment solver with test part in realistic factory setting including nominal camera orientations.

Finally, Figure 21 shows different solution boxes, including nominal camera orientations (camera oriented according to the midpoints of the orientation intervals), as well as the facets of interest valid for those specific pose solutions.

The results are extremely promising, match realistic camera imaging characteristics, and present a realistic assessment of what a real camera captures.

## 6. Conclusions

The research herein presents a realistic application of the techniques for the set-based solving of complete sets of inspection poses with additional extensions in order to account for the realistic non-linearities present in real cameras. The result is a unique solver algorithm that is able to calculate realistic pose intervals and associated visible facets, which can be certified as accurate as a result of the methods used for their evaluation. The method derived herein differs from the existing literature in that instead of trying to derive a single optimal camera deployment for a given inspection task, it generates a complete set of all possible valid poses.

The work presents novel interval constraint satisfaction algorithms for solving all 6 dimensions of pose, as well as for considering occlusions and lens distortion modelling from a set-based perspective. The result of these algorithms is a complete set of all pose intervals that satisfy the inspection task for at least one of the facets of interest. This solution set can then be used as a map of the workspace around the part in conjunction with a deployment recommendation algorithm in order to derive the recommended deployments that best satisfy the inspection deployment requirements in a real environment.

Additionally, an experimental validation was conducted that confirms that the methods do, in fact, generate results that match real imaging results and accurately predict the inspection capabilities of any given set of poses. This allows for the real-world application of these methods in generating real deployment solutions for factory environments.

Additionally, because the generated deployments are in an interval-based framework, they implicitly account for uncertainties in deployment and modelling, which allows for the further certification of the accuracy of the results of any pose box.

Future work could include improvements to the deployment recommendation algorithm and reduction of computational time for the solver, along with considerations for other sensor types. However, at this point, it is still a fully functional algorithm for the certified set-based synthesis methods for the complete set of valid inspection poses for an arbitrary 3D mesh. The methodology derived in this paper greatly reduces setup time for camera deployment and has potential applications in a wide range of industrial settings. 

## Figures and Tables

**Table 1:** Validation camera parameters.

Parameter	Value
Focal length x, $f_{x}$	2308.468 pixels
Focal length y, $f_{y}$	2307.752 pixels
Sensor width, *w*	7.12 mm
Sensor height, *h*	5.32 mm
Sensor resolution	2064 × 1544
Pixel width	3.45 μm
Pixel height	3.45 μm
Min. working distance	200 mm
$K_{1}$	−0.17900
$K_{2}$	0.11200
$K_{3}$	0.05890
$P_{1}$	0.00021
$P_{2}$	−0.00138
$c_{x}$	1007.547 pixels
$c_{y}$	792.759 pixels
$f_{stop}$	2.0

**Table 2:** Pose vectors for test poses.

Test Pose	Pose Vector
1	[−0.080, −0.300, 0.480, 3.032, 2.658, −0.087]
2	[−0.080, −0.300, 0.580, 3.207, 2.658, −0.087]
3	[−0.080, −0.200, 0.580, 3.207, 2.758, −0.087]
4	[−0.080, −0.200, 0.480, 3.032, 2.758, −0.087]
5	[0.020, −0.300, 0.480, 3.032, 2.658, 0.087]
6	[0.020, −0.300, 0.580, 3.207, 2.658, 0.087]
7	[0.020, −0.200, 0.580, 3.207, 2.758, 0.087]
8	[0.020, −0.200, 0.480, 3.032, 2.758, 0.087]

**Table 3:** Constraint parameters.

Parameter	Value
Minimum distance	0.1857 m
Maximum distance	0.9531 m
Horizontal FOV half-angle	24.1${}^{\circ }$
Vertical FOV half-angle	18.5${}^{\circ }$
Min. viewing angle	22.5${}^{\circ }$

## Data Availability

Data are contained within the article.

## Nomenclature

[X]	Interval matrix
[x]	Interval vector
[x]	Interval variable
$\hat{x}$	Unit vector
F	Constraint
L	List
P	Set
X	Matrix
x	Vector
${\left\{O\right\}}_{X}$	Coordinate frame
*x*	Scalar
